# Draft Genome of *Klebsiella pneumoniae* Sequence Type 512, a Multidrug-Resistant Strain Isolated during a Recent KPC Outbreak in Italy

**DOI:** 10.1128/genomeA.00035-12

**Published:** 2013-01-15

**Authors:** Francesco Comandatore, Paolo Gaibani, Simone Ambretti, Maria Paola Landini, Daniele Daffonchio, Piero Marone, Vittorio Sambri, Claudio Bandi, Davide Sassera

**Affiliations:** a Dipartimento di Scienze Veterinarie e Sanità Pubblica (DIVET); c Dipartimento di Scienze per gli Alimenti, la Nutrizione e l’Ambiente (DeFENS), Università degli Studi di Milano, Milan, Italy; b Unit of Clinical Microbiology, St. Orsola Malpighi University Hospital, Bologna, Italy; d Fondazione Policlinico IRCCS San Matteo, Pavia, Italy

## Abstract

Here, we present the draft genome sequence of *Klebsiella pneumoniae* subsp. *pneumoniae* sequence type 512 (ST512) isolated during a KPC-producer outbreak. This strain is resistant to β-lactams, cephalosporins, fluoroquinolones, aminoglycosides, macrolides, tetracyclines, and carbapenems but susceptible to colistin. The ST512-K30BO genome is composed of 289 contigs for 5,392,844 bp with 56.9% G+C content.

## GENOME ANNOUNCEMENT

*Klebsiella pneumoniae* is responsible for an increasing number of healthcare-related infections, mostly in patients with impaired immunity, including bloodstream and wound infections, pneumonia, and abscesses. The rapid diffusion of this pathogen is due mainly to the emergence of a number of multidrug-resistant strains (1). In particular, the first report of carbapenem-resistant *K.  pneumoniae* in 2001 was followed by a worldwide spread of different types of carbapenemase producers, including the most widespread, *K. pneumoniae* carbapenemase (KPC) and New Delhi metallo-β-lactamase (NDM) (2). The first Italian outbreak of *K.  pneumoniae* KPC producers was reported recently (3).

The *K. pneumoniae* isolate ST512-K30BO was isolated using a central venous catheter from a hospitalized patient at the St. Orsola Malpighi University Hospital in Bologna, Italy. Antimicrobial susceptibility testing was performed according to the European Committee on Antimicrobial Susceptibility Testing guidelines (4). The isolate ST512-K30BO showed multiple resistances to clinically used antibiotics, including β-lactams, cephalosporins, carbapenems, fluoroquinolones, macrolides, aminoglycosides, and tigecycline. The strain was susceptible to colistin. Whole DNA was extracted using the Qiagen DNeasy kit and subjected to quality controls. Next-generation sequencing was performed on an Illumina HiSeq 2000 platform (5) with 300-base distant paired ends. Overall, 29,008,494 paired sequences were generated, for a total of more than 5.7 gigabases and a mean length of 199 bases per pair.

The genome assembly was performed using MIRA 3.4 (6) after quality selection and trimming via a specifically designed PerlScript. The assembly was manually checked using the Gap4 software of the Staden package (7). The resulting assembly consists of 289 contigs, with a G+C content of 56.9% for a total of 5,392,844 bp. Multilocus sequence type (MLST) analysis was performed using the Center for Biological Sequence Analysis (CBS) server online tool (http://www.cbs.dtu.dk/services/MLST/). The sequenced genome was of strain 512. This strain, which is highly similar to the most widespread multidrug-resistant ST258 strain (8), has been reported previously as carbapenem-resistant and epidemic in Israel (9).

Genome annotation was performed automatically on the Rapid Annotation using Subsystem Technology (RAST) server (10) using Glimmer for base calling. Additionally, all open reading frames obtained from the RAST annotation were subjected to BLAST analysis against the Antibiotic Resistance Database (ARDB) (11) and the Comprehensive Antibiotic Resistance Database (CARD) (http://arpcard.mcmaster.ca). All of the genes indicated by at least one database as being implicated in antibiotic resistance were manually controlled. This approach highlighted the presence of 164 genes related to antibiotic resistance, including *bla*_CTX-M9_, *bla*_TEM-33_, *bla*_SHV-2_, *bla*_KPC-3_, *ant(3″)-Ia*, *ant(2″)-Ia*, *marA*, *macA*, *macB*, and *tetR*. Comparative genomic analyses will be performed to highlight similarities and differences between ST512 and other *K.  pneumoniae* strains with different antimicrobial susceptibility patterns.

### Nucleotide sequence accession number.

The genome sequence was deposited in the European Bioinformatics Institute (EBI) under accession no. CAJM01000000.
